# Conserved charged amino acid residues in the extracellular region of sodium/iodide symporter are critical for iodide transport activity

**DOI:** 10.1186/1423-0127-17-89

**Published:** 2010-11-23

**Authors:** Chia-Cheng Li, Tin-Yun Ho, Chia-Hung Kao, Shih-Lu Wu, Ji-An Liang, Chien-Yun Hsiang

**Affiliations:** 1 Graduate Institute of Chinese Medicine, China Medical University, Taichung 40402, Taiwan; 2 Department of Nuclear Medicine, China Medical University Hospital, Taichung 40447, Taiwan; 3 Department of Biochemistry, China Medical University, Taichung 40402, Taiwan; 4 Department of Radiation Therapy and Oncology, China Medical University Hospital, Taichung 40447, Taiwan; 5 Department of Microbiology, China Medical University, Taichung 40402, Taiwan

## Abstract

**Background:**

Sodium/iodide symporter (NIS) mediates the active transport and accumulation of iodide from the blood into the thyroid gland. His-226 located in the extracellular region of NIS has been demonstrated to be critical for iodide transport in our previous study. The conserved charged amino acid residues in the extracellular region of NIS were therefore characterized in this study.

**Methods:**

Fourteen charged residues (Arg-9, Glu-79, Arg-82, Lys-86, Asp-163, His-226, Arg-228, Asp-233, Asp-237, Arg-239, Arg-241, Asp-311, Asp-322, and Asp-331) were replaced by alanine. Iodide uptake abilities of mutants were evaluated by steady-state and kinetic analysis. The three-dimensional comparative protein structure of NIS was further modeled using sodium/glucose transporter as the reference protein.

**Results:**

All the NIS mutants were expressed normally in the cells and targeted correctly to the plasma membrane. However, these mutants, except R9A, displayed severe defects on the iodide uptake. Further kinetic analysis revealed that mutations at conserved positively charged amino acid residues in the extracellular region of NIS led to decrease NIS-mediated iodide uptake activity by reducing the maximal rate of iodide transport, while mutations at conserved negatively charged residues led to decrease iodide transport by increasing dissociation between NIS mutants and iodide.

**Conclusions:**

This is the first report characterizing thoroughly the functional significance of conserved charged amino acid residues in the extracellular region of NIS. Our data suggested that conserved charged amino acid residues, except Arg-9, in the extracellular region of NIS were critical for iodide transport.

## Background

Sodium/iodide symporter (NIS) is a transmembrane glycoprotein that is functionally expressed in thyroids, salivary glands, gastric mucosa, and lactating mammary glands [1]. NIS mediates the active transport of iodide into the follicular thyroid cells and, in turn, concentrates iodide in the thyroid glands. The ability of cancerous thyroid cells to actively transport iodide via NIS has provided a unique and effective delivery system for the detection and destruction of these cells with radioiodide [2].

NIS is a member of solute-sodium symporters. Solute-sodium symporters are a large family of proteins that co-transport sodium ions with sugars, amino acids, vitamins, or iodide [3,4]. So far, more than 250 members of solute-sodium symporters family have been identified, and several members, including NIS, human sodium/glucose transporter (hSGLT), *Vibrio parahaemolyticus *SGLT (vSGLT) and *Escherichia coli *(*E. coli*) proline symporter, have been well characterized [2-4]. NIS as well as other members transport sodium and solute via an alternating access mechanism with tight coupling between sodium and solute transport [5-7]. However, the absence of structural/functional data of NIS may be difficult to explain this hypothesis.

NIS mutations detected in patients with congenital iodide transport defect (ITD) have provided the significant structural/functional information about NIS. Twelve ITD-causing NIS mutations, which are situated in the transmembrane or intracellular segments of NIS, have been characterized so far: V59E, G93R, Q267E, C272X, G395R, T354P, frame-shift 515X, Y531X, G543E, ΔM143-Q323, and ΔA439-P443 [2,8]. Mutations at the highly conserved serine and threonine residues in the transmembrane segment IX have shown that Thr-351, Ser-353, Thr-354, Ser-356, and Thr-357 play a key role in sodium/iodide co-transport [9]. Phosphorylation sites (Ser-43, Thr-49, Ser-227, Thr-577, and Ser-581) of NIS have been identified to be important for NIS protein stability and function [10]. In addition, His-226 located in the extracellular region of NIS is critical for iodide transport in our previous study [11]. Moreover, deletion in the region spanning residues 233-280 of NIS loses the iodide uptake activity [12]. In this study, we elucidated the importance of 14 conserved charged amino acid residues, which were located in the extracellular region of NIS, by site-directed mutagenesis and kinetic analysis. Our findings indicated that all mutants, except R9A, displayed severe defects on the iodide uptake. Moreover, mutations at positively charged amino acid residues led to the decrease in *V*max, while mutations at negatively charged residues resulted in the increase in *K*m. Our data suggested that conserved charged amino acid residues, except Arg-9, in the extracellular region of NIS were critical for iodide transport.

## Methods

### Cloning and site-directed mutagenesis

Human NIS cDNA was cloned as described previously [11]. Briefly, two overlapping cDNA fragments representing either the 5'-half or the 3'-half of the complete NIS coding region were amplified and inserted into pBluescript^®^II KS (-) vector to create pBKS-NIS-5' and pBKS-NIS-3' plasmids, respectively. A full-length NIS clone was then constructed by in-frame fusion of both halves using a unique *Bgl *II site in the overlap of the fragments. Site-directed mutagenesis was performed as described previously [13]. Briefly, uracil-containing single-stranded DNA (ssDNA) was prepared by transforming pBKS-NIS-5' into *E. coli *CJ236 strain. Uracil-containing ssDNA was annealed with 5'-kinase primer, the second-stranded DNA was synthesized, and the double-stranded DNA was then transformed into *E. coli *NM522 strain to allow the mutated strand to be amplified. The full-length NIS mutant clones were subcloned into pcDNA3.1 expression vector (Invitrogen, SanDiego, CA) to create pcDNA3.1-NIS plasmid DNA. The primers for the construction of NIS mutants are shown in Additional File 1; Table S1. All the mutants created in this study were confirmed by sequencing (Additional File 1; Table S2).

### Cell culture and transient transfection

Human hepatoblastoma HepG2 cell line was maintained in Dulbecco's modified Eagle's medium (DMEM) (Life Technologies, Gaithersburg, MD) supplemented with 10% fetal bovine serum (HyClone, Logan. UT). HepG2 cells were transiently transfected with pcDNA3.1-NIS wild-type, pcDNA3.1-NIS mutants, pcDNA3.1, or pcDNA3.1/lacZ by SuperFect^® ^transfection reagent (Qiagen Inc., Valencia, CA). Transfected cells were then kept in a humidified incubator at 37°C with 5% CO_2 _for 24 h.

### Total RNA extraction and reverse transcription-polymerase chain reaction (RT-PCR)

RNA extraction and RT were performed as described previously [11]. RNA integrity was electrophoretically verified by both the ethidium bromide staining and the absorption ratio (OD260/OD280 > 1.95). RT mixtures were subjected to PCR to measure the mRNAs of NIS and β-actin. PCR amplification was performed with *Taq *polymerase (Promega, Madison, WI) for 20 cycles at 94°C for 45 s, 50°C for 45 s, and 72°C for 1 min. PCR primers for NIS were as follows: sense, 5'-CTCCTCCCTGCTAACGACTC-3'; antisense, 5'-CGACCACCATCATGTCCAAC-3'; PCR primers for β-actin were as follows: sense, 5'-TGACGGGGTCACCCACACTGTGCCCATCTA-3'; antisense, 5'-CTAGAAGCATTGCGGTGGACGATGGAGGG-3'.

### Western blot analysis

The cellular proteins (10 μg) were separated by 10% sodium dodecyl sulfate-polyacrylamide gel electrophoresis, and the protein bands were then transferred electrophoretically to nitrocellulose membranes. Membranes were blocked in blocking buffer (20 mM Tris-HCl, pH 7.6, 140 mM NaCl, 0.1% Tween 20, and 5% skim milk) and probed with mouse monoclonal antibody against NIS (Lab Vision, Fremont, CA) or rabbit polyclonal antibody against β-actin (Santa Cruz, Santa Cruz, CA). The bound antibody was detected with peroxidase-conjugated anti-mouse or anti-rabbit antibody followed by enhanced chemiluminescence system (Amersham, Chalfont St. Giles, Buckinghamshire, UK) and exposed by autoradiography.

### Immunofluorescent staining

HepG2 cells were seeded in 24-well plates containing sterilized coverslips, incubated at 37°C for 2 days, and transiently transfected with DNAs. One day later, cells were washed twice with phosphate-buffered saline (PBS) (137 mM NaCl, 1.4 mM KH_2_PO_4_, 4.3 mM Na_2_HPO_4_, 2.7 mM KCl, pH 7.2), fixed with 3.7% PBS-buffered formaldehyde for 30 min at room temperature, and washed three times with PBS. Coverslips were then incubated with mouse anti-NIS monoclonal antibody overnight at 4°C, washed three times with PBS, and incubated with fluorescein-conjugated goat anti-mouse IgG antibody (Jackson ImmunoResearch, West Grove, PA) for 2 h at 37°C. Coverslips were mounted and examined using a confocal microscope (Leica, Germany), with an excitation wavelength of 488 nm. Anti-NIS monoclonal antibody was against residues 625 to 643 mapping to the carboxyl terminus of human NIS.

### Iodide uptake and reporter assays

For steady-state analysis, cells were incubated for 1 h with 10.2 μCi/ml carrier-free Na^125^I in 1 ml DMEM at 37°C. For the inhibition of NIS-mediated uptake, NaClO_4_, in a final concentration of 30 μM, was included in parallel incubations. After a 1-h incubation, medium was completely removed and washed twice with 2 ml ice-cold PBS. After washing, the cells were lysed with 350 μl Triton lysis buffer (50 mM Tris-HCl, pH 7.8, 1% Triton X-100, 1 mM dithiothreitol). Radioactivities of lysates were determined by a Cobra II auto-gamma counter (Packard BioScience, Dreieich, Germany). β-Galactosidase activities of cell lysates were analyzed by mixing cell lysates with O-nitrophenyl-β-D-galactopyranoside. After a 30-min incubation at 37°C, the absorbance values of the mixtures were measured at 420 nm.

For kinetic analysis, cells were incubated for 4 min with 6.25, 12.5, 25, 50, and 100 μM NaI, and uptake reactions were determined as described aforementioned. Data were processed using the equation: *v *= (*V*max × [I])/(*K*m + [I]) + 0.0156 × [I] + 2.4588. The terms 0.0156 × [I] + 2.4588 correspond to background adjusted by least squares to the data obtained with non-transfected cells.

### Molecular modeling

The three-dimensional comparative protein structure of NIS was modeled using vSGLT (PDB ID: 3dh4) as the reference protein. Protein structure was built using SWISS-MODEL workspace [14]. 'Frankenstein's monster' approach was applied to refinement of the NIS structure [15].

### Statistical analysis

Data were presented as mean ± standard error. Student's *t *test was used for comparisons between groups. A *p *value < 0.05 was considered to be statistically significant.

## Results

### Characterization of expression and plasma membrane targeting of wild-type and mutated NIS proteins in HepG2 cells

**Figure 1:**
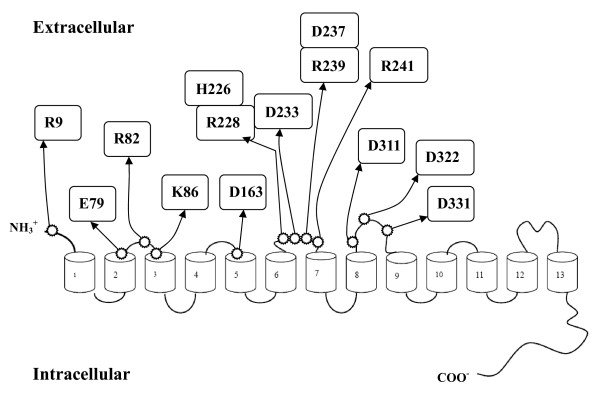
**Schematic representation of NIS secondary structure model**. The schematic diagram shows the predicted secondary structure of NIS. The commonly accepted topological model of NIS shows 13 transmembrane helices with N terminus located extracellularly and C terminus located intracellularly. Transmembrane segments are represented by cylinders. Positions of 14 amino acid residues mutated in this study are indicated by arrows.

The current NIS secondary structure model depicts NIS as a protein with 13 transmembrane segments [16]. Multiple alignments of NIS amino acid sequences from human, pig, mouse, and rat showed that 14 charged residues (Arg-9, Glu-79, Arg-82, Lys-86, Asp-163, His-226, Arg-228, Asp-233, Asp-237, Arg-239, Arg-241, Asp-311, Asp-322, and Asp-331) were highly conserved among NIS analogs (Additional File 1; Fig. S1). Additionally, all of these charged residues were located on the extracellular region of NIS (Figure 1). Therefore, 14 conserved charged residues were then replaced with noncharged amino acid, alanine, by site-directed mutagenesis.

**Figure 2:**
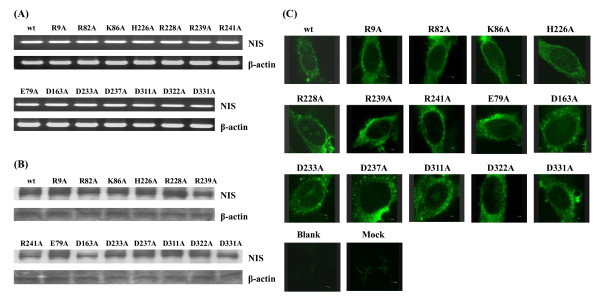
**Expression and plasma membrane targeting of NIS mutants**. (A) RT-PCR. HepG2 cells were cultured in 25-cm^2 ^flasks and transfected with wild-type (wt), R9A, E79A, R82A, K86A, D163A, H226A, R228A, D233A, D237A, R239A, R241A, D311A, D322A, or D331A plasmid DNAs. Total RNAs were extracted and 1 μg of total RNA was reverse transcribed. The resulting cDNAs were then amplified by PCR. PCR products were resolved in 1% agarose gels and visualized with ethidium bromide. (B) Western blot. HepG2 cells were cultured in 25-cm^2 ^flasks and transfected with wt or mutated NIS DNAs. The NIS and β-actin proteins in the cellular lysates were detected by Western blot. (C) Immunofluorescent staining. HepG2 cells were cultured on glass coverslips and transfected without (blank) or with pcDNA3.1 (mock), wt, or mutated NIS DNAs for 2 days. Cells were then treated with anti-NIS antibody, stained with fluorescence-conjugated secondary antibody, and evaluated under a confocal microscope. Magnification, 400×. Similar results were obtained in three independent experiments.

To verify the expression levels and plasma membrane targeting of NIS mutants, HepG2 cells were transiently transfected with wild-type or mutated NIS DNAs. Twenty-four hours later, the mRNA level, protein level, and plasma membrane targeting of NIS were evaluated by RT-PCR, Western blot, and Immunofluorescent staining, respectively. As shown in Figure 2A, no apparent difference of mRNA level was found in HepG2 cell expressing either wild type or mutants. By using mouse monoclonal antibody against the C-terminus of NIS, mutated NIS-expressing cells displayed the similar protein amount and plasma membrane-associated immunofluorescence staining pattern with wild-type NIS-expressing cells (Figures 2B and 2C). These findings indicated that NIS mutants were expressed normally in the cells and targeted correctly to the plasma membrane.

### Iodide uptake activities of NIS mutants

**Figure 3:**
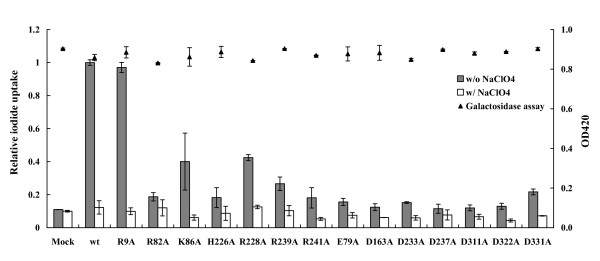
**Iodide uptake activities of NIS mutants**. HepG2 cells were transfected with pcDNA3.1/lacZ and pcDNA3.1 (mock), wild-type (wt), R9A, E79A, R82A, K86A, D163A, H226A, R228A, D233A, D237A, R239A, R241A, D311A, D322A, or D331A DNAs. Twenty-four hours later, iodide uptake abilities and β-galactosidase activities were determined as described in Materials and Methods. Iodide uptake abilities are expressed as relative iodide uptake, which is present as the comparison with the radioactivity relative to wt. β-Galactosidase activities are expressed as OD420. Values are mean ± standard error of triplicate assays.

HepG2 cells were transiently transfected with pcDNA3.1/lacZ and pcDNA3.1, wild-type, or mutated NIS DNAs. Twenty-four hours later, the iodide uptake activity was analyzed by steady-state iodide uptake assay and the transfection efficiency was monitored by β-galactosidase assay. As shown in Figure 3, wild-type NIS-expressing cells exhibited a significant highly iodide uptake activity. Perchlorate treatment led to a markedly decrease in iodide uptake, suggesting the specificity of iodide uptake assay. Mutation at Arg-9 displayed no defect on the iodide uptake activity, suggesting that Arg-9 was not involved in the iodide transport of NIS. However, replacement of other charged amino acid residues with alanine resulted in a large decrease in iodide uptake activity. β-Galactosidase activities were consistent in wild-type and mutated NIS-expressing cells, indicating that the dramatic reduced iodide uptake activities resulted from the amino acid substitution instead of transfection variation. These findings suggested that conserved charged amino acid residues, except Arg-9, in the extracellular region of NIS were critical for iodide transport.

### Kinetics analysis of NIS mutants

**Figure 4:**
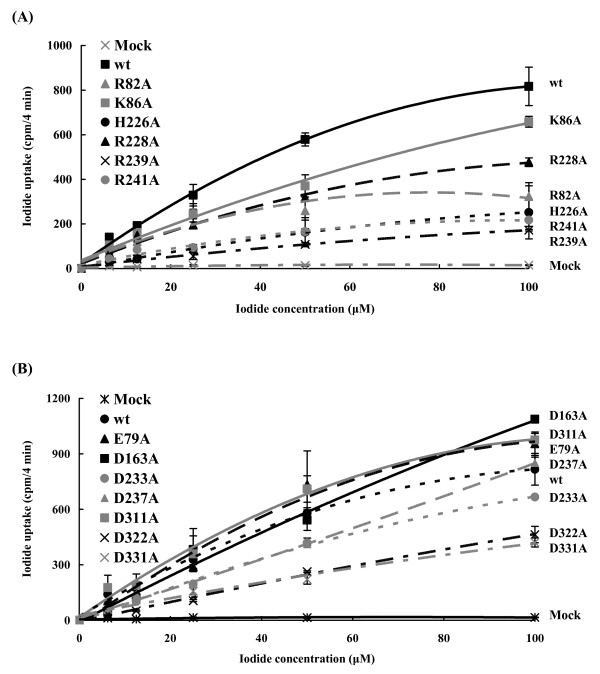
**Kinetic analysis of NIS mutants**. HepG2 cells were transfected with pcDNA3.1 (mock), wild-type (wt), or mutated NIS DNAs. After 24 h, initial rates (4 min time points) of iodide uptake were determined at the indicated concentrations of iodide. Calculated curves were generated using the equation *v *= (*V*max × [I])/(*K*m + [I]) + 0.0156 × [I] + 2.4588. The terms 0.0156 × [I] + 2.4588 correspond to background adjusted by least squares to the data obtained with non-transfected cells. Values are mean ± standard error of triplicate assays.

We further analyzed the kinetic properties of iodide uptake in HepG2 cells expressing wild-type or mutated NIS. Initial rates were assessed by measuring iodide accumulation at 4-min time points over a range of 6.25, 12.5, 25, 50, and 100 μM NaI (Figure 4). Typical Michaelis-Menten kinetic was used to determine the *V*max and *K*m values of NIS. The transfection efficiency was also monitored by β-galactosidase assay. β-Galactosidase activities were consistent in wild-type and mutated NIS-expressing cells, indicating that the transfection efficiencies were consistent in wild-type and mutants (Additional File 1; Fig. S2).A comparison of kinetic parameters for wild type and mutants is shown on Table 1. Because R9A displayed no defect on the iodide uptake activity, we did not elucidate the role of Arg-9 further. Replacement of positively charged residues (Arg-82, Lys-86, His-226, Arg-228. Arg-239, and Arg-241) by alanine resulted in a dramatic reduction in *V*max. However, mutations at negatively charged residues, except Asp-331, led to a slight change in *V*max. These findings indicated that Asp-331- and basic residues-altered mutants displayed a lower turnover rate. Replacement of Arg-239, Asp-163, Asp-233, Asp-237, and Asp-322 with alanine resulted in a significant increase in *K*m. However, mutation at Arg-82 showed a markedly decrease in *K*m. Replacement of other residues with alanine led to slight alternation in *K*m. These findings indicated that the dissociation of the Michaelis complex between mutants (R239A, D163A, D233A, D237A, and D322A) and iodide was larger than that of wild-type NIS, while the dissociation between R82A mutant and iodide was smaller than that of wild-type NIS.

**Table 1:** Kinetic analysis of human NIS mutants.

NIS mutants	*V*max^a^	*K*m^a^
Wild type	7.94 ± 0.2	81.35 ± 8.89
R82A	2.46 ± 0.31***	32.87 ± 5.58**
K86A	5.49 ± 0.61**	68.97 ± 14.08
H226A	2.02 ± 0.48***	71.37 ± 12.43
R228A	4.67 ± 0.45***	91.8 ± 7.2
R239A	2.42 ± 0.45***	171.23 ± 36.12**
R241A	2.15 ± 0.14***	67.64 ± 14.69
E79A	8.55 ± 0.46	110.42 ± 39.87
D163A	9.46 ± 3.1	166.51 ± 37.9*
D233A	8.37 ± 1.6	207.49 ± 72.65*
D237A	7.93 ± 0.52	133.46 ± 43.93*
D311A	6.37 ± 0.69	59.89 ± 16.84
D322A	9.45 ± 1.11	496.6 ± 67.35***
D331A	3.98 ± 0.72***	91.27 ± 15.32

^a ^Values are mean ± standard error of triplicate assays.

*p < 0.05, **p < 0.01, ***p < 0.001, compared with wild type.

## Discussion

Mutations at the amino acid residues in the transmembrane or intracellular segments of NIS have identified the roles of these residues on the iodide transport. For examples, mutations at Val-59 in the transmembrane segment II and Gln-267 in the intracellular loop have led to severe defects on the iodide uptake [17,18]. Mutations at the highly conserved serine and threonine residues in the transmembrane segment IX and intracellular loops have revealed that these residues play key roles in the sodium/iodide co-transport [9,10]. In addition to the amino acid residues in the transmembrane or intracellular segments, some studies have shown that extracellular loops play essential roles for the ion transport in other transporters, such as apical sodium-dependent bile acid transporter, serotonin transporter, sodium pump alpha subunit, and chloride/bicarbonate anion exchanger [19-23]. Therefore, herein we analyzed the critical roles of amino acid residues in the extracellular segments of NIS, and our findings indicated that these residues affected the iodide transport via various mechanisms.

Charged amino acid residues of some transporters have been shown to be involved in ion transport. For examples, charged residues of kidney electrogenic sodium-bicarbonate cotransporter are involved in ion recognition in putative outward-facing and inward-facing conformation [24]. Histidine residues of *E. coli *Na^+^/H^+ ^exchanger NhaA and *Arabidopsis *cation/H^+ ^exchanger are important for ion transport [22,25]. Mutation at histidine residues of Na^+^/bicarboxylate co-transporter leads to a decrease in succinate transport [26]. Histidine residues of human proton-coupled folate transporter SLC46A1 play an important role in SLC46A1 protonation [27]. Moreover, His-226 is critical for the iodide uptake activity of NIS [13]. Furthermore, arginine residues of organic anion transporter 1 influence the binding of glutarate and interact with chloride [28]. Arg-211 residue of rabbit proton-coupled peptide transporter PepT1 plays an intriguing role in the function of PepT1 [29]. In this study, we replaced the conserved charged amino acid residues with alanine and found that, except Arg-9, all the mutants displayed severe defects on iodide transport. Kinetic analysis revealed that all mutants mutated at the positively charged amino acids showed a dramatic reduction in *V*max, while most of the mutants mutated at the negatively charged residues displayed an increase in *K*m. These findings suggested that mutations at conserved basic amino acid residues in the extracellular segments of NIS led to decrease NIS-mediated iodide uptake activity by reducing the maximal rate of iodide transport, while mutations at the conserved acidic amino acid residues led to decrease iodide transport by increasing dissociation between mutants and iodide. Additionally, mutants in this study displayed reduced iodide uptake activities, suggesting that mutations at the extracellular region may lead to the lethal effect *in vivo*. This speculation may explain why NIS mutations in patients with ITD are all located in the transmembrane and intracellular segments, but not in the extracellular domain.

**Figure 5:**
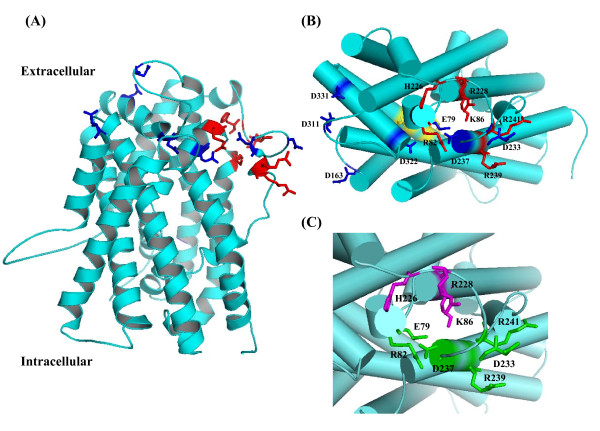
**Structure modeling of NIS**. (A) Structure modeling viewed in the membrane plane. The three-dimensional structure of NIS was modeled using vSGLT as the reference protein. Mutated residues are represented by sticks. Positively charged and negative charged residues mutated in this study are colored as red and blue, respectively. (B) Core structure viewed from the extracellular side. Residues in the transmembrane segment IX, which have been identified to be involved in sodium/iodide co-transport, are displayed as sticks and colored as yellow. (C) Close-up view of the core structure. Residues involved in the entry or binding of iodide ions are colored as green. Residues involved in the iodide transport are colored as magenta.

To explain why these conserved amino acid residues affected the iodide transport, we built the three-dimensional structure of NIS using vSGLT as a template protein. NIS has a sequence identity of 21.8% (37.6% similarity) to vSGLT (Additional File 1; Fig. S3). NIS and vSGLT are the members of solute-sodium symporters that co-transport sodium ions with sugars or iodide ions. Moreover, both share an alternating-access mechanism with tight coupling between sodium ion and solute transport [30]. The recognized homology suggested that using vSGLT as the template for the modeling of NIS is reasonable. The three-dimensional structure of NIS (residues 50-443) is shown on Figure 5. The proposed structure of NIS contained transmembrane helices in an inward-facing conformation. Amino acid residues mutated in this study were located on the extracellular segments, as expected. Interestingly, positively charged amino acid residues were situated on one side. Structure viewed from the extracellular side displayed the core structure of NIS (Figure 5B). Glu-79, Arg-82, Lys-86, His-226, Arg-228, and Asp-237 were localized around the core. Glu-79, Arg-82, and Asp-237 were localized on one side of the core. Mutations at these residues affected the *K*m values, suggesting that these amino acid residues might influence the binding of iodide ions. Lys-86, His-226, and Arg-228 were situated on the other side of the core. Mutations at these residues altered the *V*max values, suggesting that these residues might be involved in the transport of iodide ions. Asp-233, Arg-239, and Arg-241 were also situated around the core. However, the side chains of these residues were exposed to the surface. Mutations at Asp-233 and Arg-239 affected the *K*m values, suggesting that both residues might influence the entry or binding of the iodide ions. Asp-163, Asp-311, and Asp-331 were situated far from the core, and the side chain of Asp-163 was extruded into the surface. Because mutation at Asp-163 altered the *K*m dramatically, Asp-163 might affect the entry or binding of iodide ions. It is interesting to find that residues (Glu-79, Arg-82, Asp-233, Asp-237, Arg-239, and Arg-241) involved in the entry or binding of iodide ions were situated on one side of the core, while residues (Lys-86, His-226, and Arg-228) involved in the iodide transport were localized on the other side (Figure 5C). These findings suggested that iodide ions might be attracted by residues on one side of the core and then transported by residues on the other side. Previous study has shown that five hydroxyl-containing residues (Thr-351, Ser-353, Thr-354, Ser-356, and Thr-357) and Asn-360 play a key role in sodium/iodide co-transport [9]. These residues are situated along one face of transmembrane segment IX and located along the cavity might explain why these residues are critical for iodide transport.

## Conclusions

In conclusion, we have characterized the roles of 14 conserved charged amino acid residues located in the extracellular regions of NIS. We have shown that mutation at these charged amino acid residues, except Arg-9, led to the severe defects on the iodide uptake. Moreover, kinetic analysis has shown that mutations at positively charged residues led to decrease iodide uptake activity by reducing the maximal rate of iodide transport, while mutations at negatively charged residues led to decrease iodide transport by increasing dissociation between mutants and iodide. This is the first report characterizing thoroughly the functional significance of conserved charged amino acid residues in the extracellular region of NIS. Additional structural data are required to elucidate the complete mechanism of iodide transport of NIS.

## Competing interests

The authors declare that they have no competing interests.

## Authors' contributions

CCL and TYH performed the experiments on the mutagenesis, iodide uptake, kinetic, and molecular modeling. CHK participated in the design of this study and interpretation of data. SLW carried out the mutagenesis and drafted this manuscript. JAL participated in the design of this study. CYH conceived of this study, participated in its design and coordination, and drafted this manuscript. All authors read and approved the final manuscript.

## Supplementary Material

Additional file 1 **Supplementary Information**. *Table S1: *DNA oligonucleotides for the construction of human NIS mutants. *Table S2: *Sequencing analysis of NIS mutants. *Figure S1: *Multiple alignments of NIS homologs. Amino acid sequences of NIS from mouse, rat, and pig were aligned with those of human by ClustalW http://www.ebi.ac.uk. Residues that are identical in all NIS homologs are indicated by asterisks. Residues that are located on the extracellular region are highlighted in grey. Amino acid residues mutated in this study are indicated in red. *Figure S2: *β-Galactosidase activities of NIS mutants. HepG2 cells were transfected with pcDNA3.1 (mock), wild-type (wt), or mutated NIS DNAs. After 24 h, β-galactosidase activities were determined as described in Materials and Methods. β-Galactosidase activities are expressed as OD420. Values are mean ± standard error of triplicate assays. *Figure S3: *Amino acid sequence alignment and secondary structure of human NIS. Amino acid sequences of NIS were aligned with those of vSGLT by ClustalW. Residues that are identical in both proteins are indicated by asterisks. Amino acid residues mutated in this study are highlighted in red. The α-helices of vSGLT are indicated by arrows. The dashed lines represent amino acid segments that were not visualized in the crystal structure of vSGLT. Click here for file
