# Supplementary material for: Conserved charged amino acid residues in the extracellular region of sodium/iodide symporter are critical for iodide transport activity
Source: J Biomed Sci. 2010 Nov 23;17(1):89. doi: 10.1186/1423-0127-17-89 (PMC3002920; doi:10.1186/1423-0127-17-89)
Supplement: Additional file 1 — Supplementary Information. Table S1: DNA oligonucleotides for the construction of human NIS mutants. Table S2: Sequencing analysis of NIS mutants. Figure S1: Multiple alignments of NIS homologs. Amino acid sequences of NIS from mouse, rat, and pig were aligned with those of human by ClustalW http://www.ebi.ac.uk. Residues that are identical in all NIS homologs are indicated by asterisks. Residues that are located on the extracellular region are highlighted in grey. Amino acid residues mutated in this study are indicated in red. Figure S2: β-Galactosidase activities of NIS mutants. HepG2 cells were transfected with pcDNA3.1 (mock), wild-type (wt), or mutated NIS DNAs. After 24 h, β-galactosidase activities were determined as described in Materials and Methods. β-Galactosidase activities are expressed as OD420. Values are mean ± standard error of triplicate assays. Figure S3: Amino acid sequence alignment and secondary structure of human NIS. Amino acid sequences of NIS were aligned with those of vSGLT by ClustalW. Residues that are identical in both proteins are indicated by asterisks. Amino acid residues mutated in this study are highlighted in red. The α-helices of vSGLT are indicated by arrows. The dashed lines represent amino acid segments that were not visualized in the crystal structure of vSGLT. [file 1423-0127-17-89-S1.DOC]

**Supplemental Table S1: DNA oligonucleotides for the construction of human NIS mutants.**

| **Primer name** | **Primer sequence^a^** |
| --- | --- |
| R9A | 5'-ACCGGGGAG**GCC**CCCACCTTCGGAGCCTGGG-3' |
| E79A | 5'-GTGCCGTCC**GCG**GCCTATCGCTATGGCCTC-3' |
| R82A | 5'-GAGGCCTAC**GCG**TATGGCCTCAAGTTCCTCTGG-3' |
| K86A | 5'-CGCTATGGTCTC**GCG**TTCCTCTGGATGTGCCTGG-3' |
| D163A | 5'-GACCGGGCTA**GCC**ATCTGGGCGTCGCTCCTGTCC-3' |
| H226A | 5'-CCAGAAC**GCG**TCCCGGATCAACCTCATGG-3' |
| R228A | 5'-CAGAACCACTCC**GCC**ATCAACCTCATGGAC-3' |
| D233A | 5'-ACCTCATG**GCA**TTCAACCCTGACCCGAGGAGCCGC-3' |
| D237A | 5'-CTTTAACCCG**GCG**CCGAGGAGCCGCTATACATTCTGG-3' |
| R239A | 5'-CCTGACCCG**GCT**AGCCGCTATACATTCTGGAC-3' |
| R241A | 5'-CGAGGAGC**GCG**TATACATTCTGGACTTTTGTGG-3' |
| D311A | 5'-CTGACTGC**GCA**CCTCTCCTCCTGGGGCGC-3' |
| D322A | 5'-TCTGCCCCT**GCG**CAGTACATGCCTCTGCTGG-3' |
| D331A | 5'-GCTGGTGCTA**GCC**ATCTTCGAAGATCTGCCTGG-3' |

^a^ Bold sequences indicate codon changes

**Supplemental Table S2: Sequencing analysis of NIS mutants.**

| **Mutants** | **Sequencing data** |
| --- | --- |
| R9A | 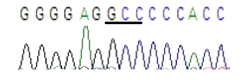 |
| E79A | 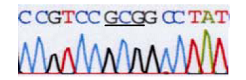 |
| R82A | 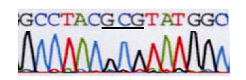 |
| K86A | 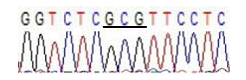 |
| D163A | 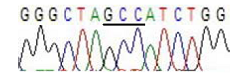 |
| H226A | 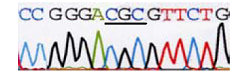 |
| R228A | 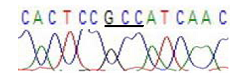 |
| D233A | 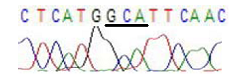 |
| D237A | 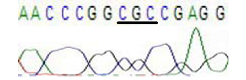 |
| R239A | 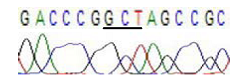 |
| R241A | 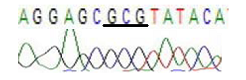 |
| D311A | 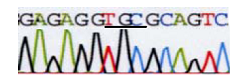 |
| D322A | 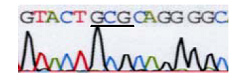 |
| D331A | 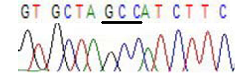 |

**Supplemental figures**

**Supplemental Figure S1: Multiple alignments of NIS homologs.** Amino acid sequences of NIS from mouse, rat, and pig were aligned with those of human by ClustalW (http://www.ebi.ac.uk). Residues that are identical in all NIS homologs are indicated by asterisks. Residues that are located on the extracellular region are highlighted in grey. Amino acid residues mutated in this study are indicated in red.

**Supplemental Figure S2: β-Galactosidase activities of NIS mutants.** HepG2 cells were transfected with pcDNA3.1 (mock), wild-type (wt), or mutated NIS DNAs. After 24 h, β-galactosidase activities were determined as described in Materials and Methods. β-Galactosidase activities are expressed as OD420. Values are mean ± standard error of triplicate assays.

**Supplemental Figure S3: Amino acid sequence alignment and secondary structure of human NIS.** Amino acid sequences of NIS were aligned with those of vSGLT by ClustalW. Residues that are identical in both proteins are indicated by asterisks. Amino acid residues mutated in this study are highlighted in red. The α-helices of vSGLT are indicated by arrows. The dashed lines represent amino acid segments that were not visualized in the crystal structure of vSGLT.

**Supplemental Figure S1.**

Human MEAVETGERPTFGAWDYGVFALMLLVSTGIGLWVGLARGGQRSAEDFFTGGRRLAALPVGLSLSASFMSAVQVLGVPSEAYRYGLKFLWM 120

Mouse MEGAEAGARATFGPWDYGVFATMLLVSTGIGLWVGLARGGQRSADDFFTGGRQLAAVPVGLSLAASFMSAVQVLGVPAEAARYGLKFLWM 120

Rat MEGAEAGARATFGAWDYGVFATMLLVSTGIGLWVGLARGGQRSADDFFTGGRQLAAVPVGLSLAASFMSAVQVLGVPAEAARYGLKFLWM 120

Pig MATVEGGARATFGAWDYGVFALMLLVSTGIGLWVGLARGGQRSAEDFFTGGRRLTAVPVGLSLSASFMSAVQVLGVPAEAYRYGLKFLWM 120

* * * * *** ******* ********************** ******* * * ****** ************* ** *********

Human CLGQLLNSVLTALLFMPVFYRLGLTSTYEYLEMRFSRAVRLCGTLQYIVATMLYTGIVIYAPALILNQVTGLDIWASLLSTGIICTFYTA 180

Mouse CVGQLLNSLLTALLFLPIFYRLGLTSTYQYLELRFSRAVRLCGTLQYLVATMLYTGIVIYAPALILNQVTGLDIWASLLSTGIICTLYTT 180

Rat CAGQLLNSLLTAFLFLPIFYRLGLTSTYQYLELRFSRAVRLCGTLQYLVATMLYTGIVIYAPALILNQVTGLDIWASLLSTGIICTLYTT 180

Pig CLGQLLNSLLTALLFLPVFYRLGLTSTYQYLELRFSRAVRLCGTLQYLVATMLYTGVVIYAPALILNQVTGLDIWASLLSTGIICTFYTT 180

* ****** *** ** * ********** *** ************** ******** ***************************** **

Human VGGMKAVVWTDVFQVVVMLSGFWVVLARGVMLVGGPRQVLTLAQNHSRINLMDFNPDPRSRYTFWTFVVGGTLVWLSMYGVNQAQVQRYV 270

Mouse VGGMKAVVWTDVFQVVVMLVGFWVILARGVMLMGGPWNVLSLAQNHSRINLMDFDPDPRSRYTFWTFVVGGSLVWLSMYGVNQAQVQRYV 270

Rat VGGMKAVVWTDVFQVVVMLVGFWVILARGVILLGGPRNVLSLAQNHSRINLMDFDPDPRSRYTFWTFIVGGTLVWLSMYGVNQAQVQRYV 270

Pig VGGMKAVIWTDVFQVLVMLTGFWVVLARGTVLVGGPGRVLELAKNHSRINLMDFDLDPRRRYTFWTFVVGGTLVWLSMYGVNQAQVQRYV 270

******* ******* *** **** **** * *** ** ** ********** *********** *** ******************

Human ACRTEKQAKLALLINQVGLFLIVSSAACCGIVMFVFYTDCDPLLLGRISAPDQYMPLLVLDIFEDLPGVPGLFLACAYSGTLSTASTSIN 360

Mouse ACHTERKAKLALLVNQLGLFLIVASAACCGIVMFVYYKDCDPLLTGRIAAPDQYMPLLVLDIFEDLPGVPGLFLACAYSGTLSTASTSIN 360

Rat ACHTEGKAKLALLVNQLGLFLIVASAACCGIVMFVYYKDCDPLLTGRISAPDQYMPLLVLDIFEDLPGVPGLFLACAYSGTLSTASTSIN 360

Pig ACRTEKQAKLALLINQVGLFLIVSSAAACGIVMFALYVDCDPLLAGHISAPDQYMPLLVLDIFEDLPGVPGLFLACAYSGTLSTASTSIN 360

** ** ****** ** ****** *** ** **** * ****** * * *****************************************

Human AMAAVTVEDLIKPRLRSLAPRKLVIISKGLSLIYGSACLTVAALSSLLGGGVLQGSFTVMGVISGPLLGAFILGMFLPACNTPGVLAGLG 450

Mouse AMAAVTVEDLIKPRMPSLAPRKLVFISKGLSFIYGSTCLTVAALSSLLGGGVLQGSFTVMGVISGPLLGAFTLGMLLPACNTPGVLSGLT 450

Rat AMAAVTVEDLIKPRMPGLAPRKLVFISKGLSFIYGSACLTVAALSSLLGGGVLQGSFTVMGVISGPLLGAFTLGMLLPACNTPGVLSGLA 450

Pig AMAAVTVEDLIKPRLPNLAPRRLVIISKGLSLIYGSACLTVAALSSLLGGGVLQGSFTVMGVISGPLLGAFVLGMFLPSCNTSGVLSGLA 450

************** **** ** ****** **** ********************************** *** ** *** *** **

Human AGLALSLWVALGATLYPPSEQTMRVLPSSAARCVALSVNASGLLDPALLPANDSSRAPSSGMDASRPALADSFYAISYLYYGALGTLTTV 540

Mouse AGLAVSLWVAVGATLYPPGEQTMGVLPTSAAGCT----NASVLPSP-PGAANTSRGIPSSGMDSGRPAFADTFYAVSYLYYGALGTLTTM 535

Rat AGLAVSLWVAVGATLYPPGEQTMGVLPTSAAGCT----NDSVLLGP-PGATNASNGIPSSGMDTGRPALADTFYAISYLYYGALGTLTTM 535

Pig AGLALSLWVAVGASLYPPSAQSMGVLPSSAAGCALPTANASGLQDP-VLAVNASSTASSLETDPEQPILAASFYAISYLYYGALGTLSTI 539

**** ***** ** **** * * *** *** * * * * * * * * * * * *** *********** *

Human LCGALISCLTGPTKRSTLAPGLLWWDLARQTASVAPKEEVAILDDNLVK-GPEELPTGNKKPPGFLPTNEDRLFFLGQKELEGAGSWTPC 629

Mouse LCGALISYLTGPTKRSSLGPGLLWWDLARQTASVAPKEDTTTLEDSLVK-GPEDIPAATKKPPGFRPEAETHPLYLG------------- 611

Rat LCGALISYLTGPTKRSSLGPGLLWWDLARQTASVAPKEDTATLEESLVK-GPEDIPAVTKKPPGLKPGAETHPLYLG------------- 611

Pig LCGALISCLTGPTKRSALGPGLLWWDLTRQTASVAPKEEVAALDDSLMKQGAEELPLAIKKPPDFLSTNEDHLLFLGQKEVNGASSKTPG 629

******* ******** * ******** ********** * * * * * * **** * **

Human VGHDGGRDQQETNL 643

Mouse --HD-----VETNL 618

Rat --HD-----VETNL 618

Pig SEHDKGHDLRETDL 643

** ** *

**Supplemental Figure S2.**

**Supplemental Figure S3.**
